# Hospital-Based Nurses’ Perceptions of the Adoption of Web 2.0 Tools for Knowledge Sharing, Learning, Social Interaction and the Production of Collective Intelligence

**DOI:** 10.2196/jmir.1398

**Published:** 2011-11-11

**Authors:** Adela S.M Lau

**Affiliations:** ^1^Interdisciplinary Programs OfficeThe Hong Kong University of Science and TechnologyHKUSTHong KongChina

**Keywords:** E-learning & Collective Intelligence, Web 2.0 tools, human behavioral adoption

## Abstract

**Background:**

Web 2.0 provides a platform or a set of tools such as blogs, wikis, really simple syndication (RSS), podcasts, tags, social bookmarks, and social networking software for knowledge sharing, learning, social interaction, and the production of collective intelligence in a virtual environment. Web 2.0 is also becoming increasingly popular in e-learning and e-social communities.

**Objectives:**

The objectives were to investigate how Web 2.0 tools can be applied for knowledge sharing, learning, social interaction, and the production of collective intelligence in the nursing domain and to investigate what behavioral perceptions are involved in the adoption of Web 2.0 tools by nurses*.*

**Methods:**

The decomposed technology acceptance model was applied to construct the research model on which the hypotheses were based. A questionnaire was developed based on the model and data from nurses (n = 388) were collected from late January 2009 until April 30, 2009. Pearson’s correlation analysis and *t* tests were used for data analysis.

**Results:**

Intention toward using Web 2.0 tools was positively correlated with usage behavior (*r* = .60, *P* < *.05).* Behavioral intention was positively correlated with attitude (r = .72, *P* < .05), perceived behavioral control (*r* = .58, *P* < .05), and subjective norm (*r* = .45, *P* < *.05*). In their decomposed constructs, perceived usefulness (*r* = .7, *P* < .05), relative advantage (*r* = .64, *P* < .05), and compatibility (*r* = .60*,* *P* < .05) were positively correlated with attitude, but perceived ease of use was not significantly correlated (*r* = .004, *P* < .05) with it. Peer (*r* = .47, *P* < .05), senior management (*r* = .24*,* *P* < .05), and hospital (*r* = .45, *P* < .05) influences had positive correlations with subjective norm. Resource (*r* = .41*,* *P* < .05) and technological (*r* = .69*,* *P* < .05) conditions were positively correlated with perceived behavioral control.

**Conclusions:**

The identified behavioral perceptions may further health policy makers’ understanding of nurses’ concerns regarding and barriers to the adoption of Web 2.0 tools and enable them to better plan the strategy of implementation of Web 2.0 tools for knowledge sharing, learning, social interaction, and the production of collective intelligence.

## Introduction

Web 2.0 tools are people-based knowledge sharing, learning, social interaction, and collective intelligence tools that support knowledge collaboration, exchange, sharing, and creation. They provide the platform and tools such as blogs, wikis, podcasts, social bookmarks, really simple syndication (RSS), tags, and social networking software to enable learners to interact and communicate in a virtual environment [1,2]. Following the rapid growth in usage of Web 2.0 tools in knowledge sharing, learning, social interaction, and the production of collective intelligence [1,3-7], this paper aimed to investigate how Web 2.0 tools are to be applied in the nursing domain for these purposes and to investigate the behavioral perceptions of the adoption of Web 2.0 tools by nurses. The objectives of this study were to investigate how Web 2.0 tools can be applied for knowledge sharing, learning, social interaction, and the production of collective intelligence; to design a research model to identify factors influencing nurses’ intention to adopt the tool; to design hypotheses and a questionnaire based on the model; and to collect the data and identify the factors influencing nurses’ intention to adopt Web 2.0 tools for knowledge sharing, learning, social interaction, and the production of collective intelligence.

In the following sections, the use of Web 2.0 tools for the purposes mentioned above is discussed. The human adoption behavior models are reviewed and the proposed model, hypotheses, and questionnaire are designed. The sampling and statistical techniques used are also presented as well as the pilot testing and data collection results. Finally, the implications of the results and conclusions are discussed.

### How Web 2.0 Tools Support Knowledge Sharing, Learning, Social Interaction, and the Production of Collective Intelligence by Nurses

Web 2.0 technologies such as blogs, wikis, really simple syndication, podcasts, tags, social bookmarks, and social networking software have the features of social interaction and collaboration to facilitate knowledge sharing, learning, social interaction, and the production of collective intelligence over the Internet [8,9]. Web 2.0 technologies allow a community to publish and edit a document collaboratively in a virtual environment [10]. Through such social interaction and collective intelligence, knowledge is created, exchanged, and shared.

#### Blog

A blog is a user-friendly content management tool that allows users (bloggers) to publish their own content on the Web [1,2,11-14]. A blogger shares his or her writings (blogs), gains comments or opinions from other bloggers, and links his or her blog to other blogs. Through such blog sharing and linkage, communities with the same interests and discussion topics are formed. Using blogs, nurses can learn about workplace experiences from each other, helping them to gain nursing knowledge from the virtual community and via social interaction [8].

#### Wiki

A wiki is a collaborative editing tool that allows authors to coedit a document [2,10-12,14,15]. A wiki has the features of content management, versioning control, rights management, and so forth [8]. Authors collaboratively edit, review, and revise a single document. Through such collaboration and collective intelligence, knowledge is created and acquired. Using a wiki, nurses can go through collaborative and reflective learning processes to gain knowledge from other nurses and apply this knowledge to solving a problem.

#### Really Simple Syndication

Really simple syndication (RSS) is a feed reader for content distribution, dissemination, and acquisition over Internet sources [14]. The RSS feed reader automatically sends an alert signal and pushes the updated content to RSS subscribers so that they can gather the most up-to-date information in real time. Using the RSS, nurses can share Internet resources with others to facilitate knowledge sharing, updating, and learning in a real-time environment.

#### Podcast

A podcast is a series of audio or video digital media files for playback on portable media players and computers [1,11,16]. It can be syndicated, subscribed to, and downloaded automatically when the content is updated. Podcasters distribute and disseminate digital media files over the Internet, and subscribers can obtain podcasts via an RSS feed reader at any time [8]. Using RSS, nurses can share or capture nursing skills and techniques in image, audio, or video files with other nurses via RSS to enable nursing learning and production of collective intelligence to take place anytime and anywhere.

#### Tags

Tags are the keywords or terms for describing digital media content such as social bookmarks, audio clips, video clips, blogs, wikis, and websites. Tags are built by a community and are used to describe its content [8]. The tag cloud function collects and counts the number of tags used by a community and groups and classifies them into different topics that enable a search engine to search more accurately [17]. Nurses can tag websites or learning resources for sharing.

#### Social Bookmark

A social bookmark enables Internet users to store, organize, search, and manage webpage bookmarks [2,17] and is described by tags. By clustering the bookmark’s tags, bookmark pages can be linked and clustered into different topics. Nurses can use social bookmarks for knowledge sharing and learning, to shorten their resource searching costs, and to facilitate the social learning atmosphere by sharing resources.

#### Social Networking Software

Social networking software typically provides social networking functions such as audio/video conferencing, Internet protocol (IP) telephony, desktop sharing, chat rooms, and whiteboards to enable a community to communicate and interact in a virtual environment. Professional social networking software may provide community-building functions such as an electronic portfolio, resume builder, and social networking so that people can be connected together to form online communities to exchange and share knowledge [8]. Using social networking software, nurses can build and maintain their social community, thereby facilitating social interaction, learning, and the production of collective intelligence over the Internet, in a similar way as patients are doing [18,19].

In summary, Web 2.0 tools provide the features of collaborative work, social networking, community, and self-management. By using social networking software, blogs, and wikis, nurses can build communities and learn through knowledge collaboration, exchange, and sharing [7]. Web 2.0 tools provide a networked environment for learners to interact with each other in a single place and to learn new knowledge through social interaction and reflective learning processes. RSS, podcasts, tags, and social bookmarks are some other Web 2.0 tools that link up Internet learning resources in a virtual, distributed, and real-time environment that facilitates knowledge sharing and learning. However, the attitude of nurses to the adoption of Web 2.0 tools is critical to the success of its application for knowledge sharing, learning, and social interaction. What are the behavioral perceptions influencing nurses in the adoption of Web 2.0 tools? Since hospital-based nurses may require more collaboration, interaction, and knowledge sharing on patient care and nursing diagnosis than non–hospital-based nurses, this study mainly focuses on surveying hospital-based nurses.

### Human Behavior Models

**Figure 1:**
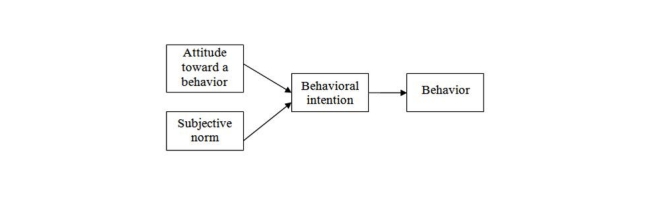
Theory of reasoned action.

**Figure 2:**
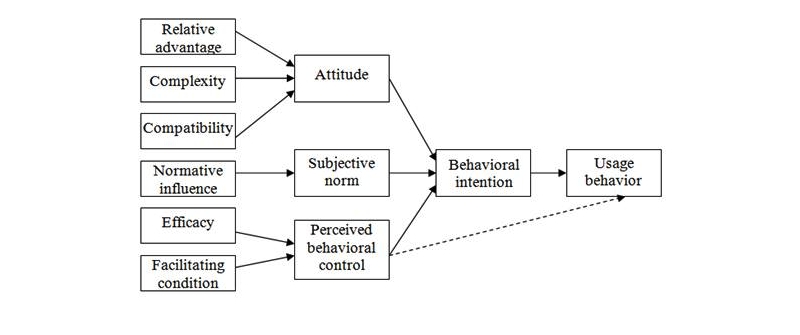
Theory of planned behavior.

The theory of reasoned action (TRA), the theory of planned behavior (TPB) [20] and the technology acceptance model (TAM) [21,22] are the most widely used human behavior models [23,24,25] for studying human perceptions of the adoption of behaviors. The TRA (see Figure 1) predicts and explains the causes of behavior by evaluating a person’s attitude and subjective norms [26,27]. The TPB (see Figure 2) is similar to what is advocated by the theory of reasoned action (TRA) but with the injection of perceived behavioral control [28] in which personal beliefs such as resources, opportunities, and obstacles are considered. In other words, the TPB studies not only the perceptions of social individual variables but also internal and external constraints on the behavior.

**Figure 3:**
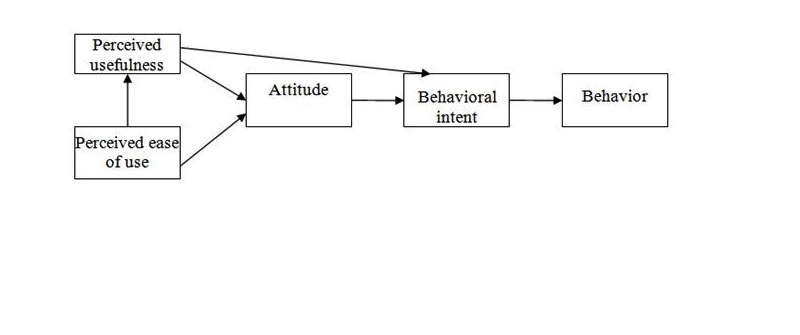
Technology acceptance model.

However, human behavior with regard to adoption of information technology (IT) cannot be described by these social individual variables and constraints alone. Human behavior may involve some practical concerns or facilitating conditions. Thus, the TAM (see Figure 3) was developed by Davis to explain computer usage behavior [29,30] and is more oriented to analyzing human behavior with regard to IT than the TRA and TPB [31]. The two attributes, perceived usefulness and ease of use [30], determine major external variables that may affect the human decision to use IT. In turn, they form the actual outcome of an action. However, subjective norm is abandoned in this model due to “its uncertain theoretical and psychometric status” [29]. In addition, perceived behavioral control is also omitted from the TAM.

**Figure 4:**
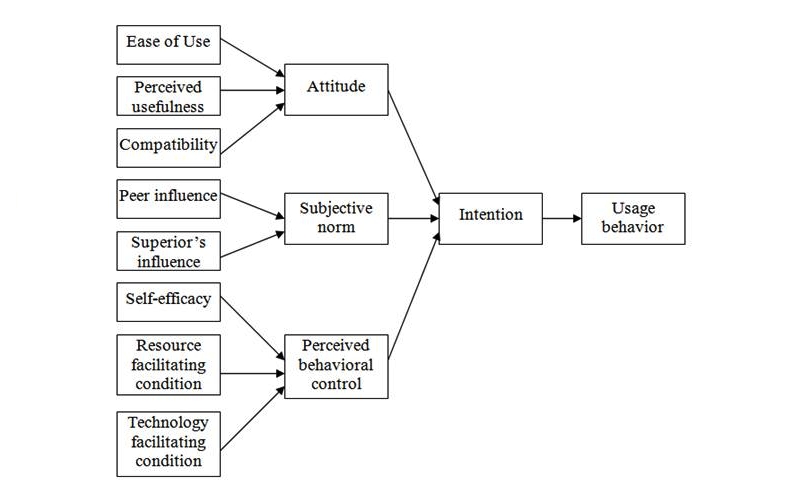
Decomposed theory of planned behavior.

Thus, the decomposed theory of planned behavior (DTPB) [32-34] is derived from the basic beliefs and structure of the theory of planned behavior model. In the DTPB model (see Figure 4), attitude, subjective norm, and perceived behavior control are further decomposed into smaller constructs. This provides a more comprehensive explanation of adoption behavior. It has been said that “the model becomes more managerially relevant, pointing to specific factors that may influence adoption and usage.” This DTPB model also takes advantage of TAM, as it identifies specific salient beliefs that may influence IT usage. It incorporates significant subfactors, including relative advantage, compatibility, normative influence (subjective norm), efficacy, and facilitating condition, which are important determinants of human behavior.

The DTBP is more managerially relevant—pointing to specific factors that may influence adoption and usage—and is more understandable as a result of focusing on specific factors of the technology acceptance research context. Thus, the DTBP was used as the framework of the research model to study nurses’ behavioral perceptions on Web 2.0 tools adoption. Details of the proposed model are discussed in the following sections.

### Proposed Model

**Figure 5:**
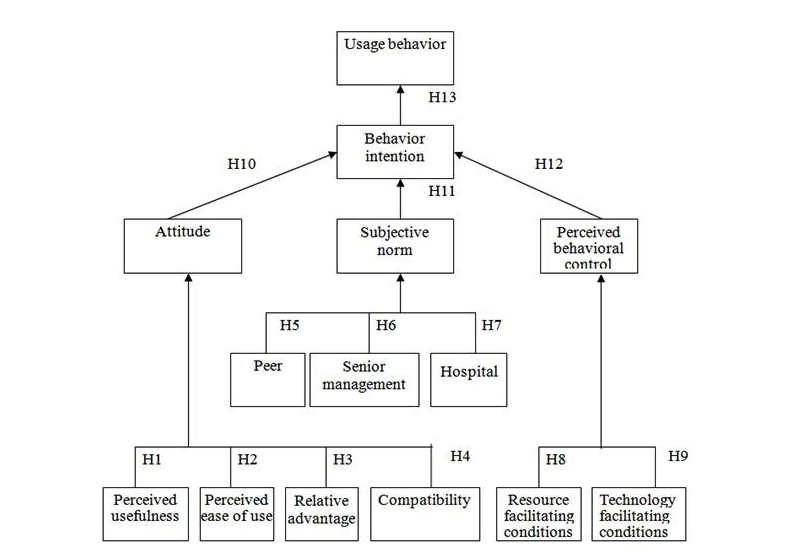
Proposed model for studying factors influencing the adoption of Web 2.0 tools.

Based on the DTPB model, a new proposed theoretical framework was established for studying the adoption of Web 2.0 tools among Hong Kong nurses. The proposed model and model description (see Figure 5 and Table 1) for studying factors influencing the adoption of Web 2.0 tools among Hong Kong nurses are demonstrated below. The usage behavior of adopting Web 2.0 tools is determined by behavioral intention, and the three major determinants—attitude, subjective norm, and perceived behavioral control—are used to determine the behavioral intention. The three major determinants are further decomposed into detailed belief constructs. Perceived usefulness, perceived ease of use, relative advantage, and compatibility are the constructs that determine attitude to Web 2.0 tools. Peers, senior management, and hospital influences are the constructs that determine subjective norm. Resources and technology-facilitating conditions are the constructs that determine perceived behavioral control. 

Based on the model, the hypotheses were set (see Table 2) and the questionnaire developed (see Multimedia Appendix 1).

**Table 1:** Model description

Construct	Description
Behavior or usage behavior (UB)	A person’s performance of a specific action or an individual’s decision to use Web 2.0 tools
Behavioral intention	A measure of the strength of intention to perform a specific action
Attitude	Whether a person possesses positive or negative feelings toward the behavior he or she performs
Perceived usefulness	The degree to which a person believes that using a particular system would enhance his or her job performance
Compatibility	The degree to which the innovation fits with the potential adopter's existing values, previous experiences, and current needs
Perceived ease of use	The degree to which a person believes that using Web 2.0 tools will be free of effort
Relative advantage	The degree to which an innovation is perceived as better than the idea it supersedes
Subjective norm	The perceived social pressure to perform a behavior
Peer influence, senior management influence, hospital influence	Influence of significant referents in our case
Perceived behavior control	The perception of the availability of skills, resources, and opportunities
Resource facilitating conditions	Resource factors such as time, money, and other factors relating to technology compatibility issues
Technology facilitating conditions	Available technology that is needed to make use of Web 2.0 tools

**Table 2:** Hypothesis setting

Hypothesis Number	Statement of Hypothesis	Question Number(s)
H1	Perceived usefulness of using Web 2.0 tools is positively correlated with attitude toward its adoption.	10, 11
H2	Perceived ease of use of Web 2.0 tools is positively correlated with attitude toward its adoption.	1, 2, 3
H3	Relative advantage of using Web 2.0 tools is positively correlated with attitude toward its adoption.	4, 5, 8
H4	Compatibility of using Web 2.0 tools is positively correlated with attitude toward its adoption.	6, 7, 9
H5	Peers’ attitude toward using Web 2.0 tools is positively correlated with subjective norm.	16
H6	Senior management’s attitude toward using Web 2.0 tools is positively correlated with subjective norm.	15
H7	Hospital’s attitude toward using Web 2.0 tools is positively correlated with subjective norm.	17
H8	Resource facilitating conditions of Web 2.0 tools are positively correlated with perceived behavioral control.	18
H9	Technology facilitation conditions for using Web 2.0 tools are positively correlated with perceived behavioral control.	20
H10	Attitude toward Web 2.0 tools adoption is positively correlated with behavioral intention.	14
H11	Subjective norm concerning Web 2.0 tools adoption is positively correlated with behavioral intention.	19
H12	Perceived behavioral control of Web 2.0 tools adoption is positively correlated with behavioral intention.	21
H13	Behavioral intention of Web 2.0 tools adoption is positively correlated with usage behavior.	12, 13

## Methods

### Sampling

There were 19,068 qualified nursing staff members in public hospitals during the fiscal year 2007-2008. With a confidence level of 95% and a confidence interval of 5, under the 50% preference, the required sample size was 377. Full-time qualified frontline registered nurses who were working under private and public hospitals and providing nursing care were included in this study. Enrolled and registered nurses who were working in outpatient departments, daycare centers, and the operating theater were excluded from this study.

### Ethics Approval and Data Access

Ethics approval was obtained from the Research Approval Committee of the Hong Kong Polytechnic University. Data access in this study was approved by the nursing research approval committees of the Caritas Medical Centre, the School of Nursing at the Hong Kong Polytechnic University, and the Nethersole School of Nursing at the Chinese University of Hong Kong from late January 2009 until April 2009 when random sampling of qualified subjects was performed. 

### Data Collection Procedures

The purpose, nature, benefits, and risks of the study and the data collection procedures were explained to the subjects. Consent was obtained from all subjects involved in this survey.

The questionnaires were distributed and collected by the general nursing manager of the hospital. The return of the questionnaire was on a voluntary basis in a sealed envelope so as to ensure anonymity and confidentiality as stated in the cover letter of each questionnaire. The questionnaires for the sample recruited at the Hong Kong Polytechnic University and the Chinese University of Hong Kong were distributed by email and in person. The return of the questionnaire was also on a voluntary basis, with consents given by subjects and anonymity and data confidentiality being similarly ensured.

### Statistical Analysis Methods

Pearson’s correlation coefficient, *r*, and *t* test were used [35]. The correlation coefficient was used to study the strength of relationship between two constructs and the *t* test was used to determine whether the correlation itself was due to chance or not (ie, the significance level of the correlation). 

## Results

### Pilot Testing

As a pilot test, the draft questionnaire was distributed to 34 nurses in the Hong Kong Polytechnic University, and 30 nurses returned them. The sample subjects found all the questions to be clear and understandable. No revisions were required. Only the reason for having nurses adopt Web 2.0 tools was found to be unclear. This aspect was then modified.

### Response Rate of Sample

To meet the calculated sample size of 377, a total of 1053 questionnaires were distributed, and 392 questionnaires were returned. Of these, 4 had not been completed, leaving 388 questionnaires for analysis for a response rate of 37%. 

### Demographic Characteristics of the Sample

The demographic characteristics of the sample are presented below (Table 3). The average age of the respondents was young, with the majority less than 30 years of age. Of the 388 respondents, 56% (219) were 21 to 30 years of age, 29% (111) were 31 to 40 years of age, and 14% (53) were 41 to 50 years of age. Only 1% (5) of respondents were 51 to 60 years of age, and none was over 60 years of age. Also, of the 388 respondents, 81% (314) were female and 19% (74) were male, while 66% (256) were single, 33% (129) were married, and 1% (3) were divorced.

In terms of education level, 26% (101/388) were subdegree holders (diploma), 64% (248/388) were degree holders, and 10% (39/388) had received a master’s level education. Most respondents were receiving continuous education (69% or 266/388) and clinical training (56% or 216/388). Almost all respondents were in good physical health, defined as having no or only one medical problem (89% or 344/388). Again, of the 388 respondents, 91% (354) were registered nurses and 9% (34) were advanced practice nurses. The majority of the 388 respondents had more than 5 years’ working experience (56% or 216).

**Table 3:** Demographic characteristics of the sample (n = 388)

Characteristics		Frequency (%)
Age group	21-30	219 (56%)
	31-40	111 (29%)
	41-50	53 (14%)
	51-60	5 (1%)
	>60	0 (0%)
Gender	Female	314 (81%)
	Male	74 (19%)
Marital status	Single	256 (66%)
	Married	129 (33%)
	Divorced	3 (1%)
Educational level	Sub-degree	101 (26%)
	Bachelor’s	248 (64%)
	Master’s	39 (10%)
Continuous education	No	122 (31%)
	Yes	266 (69%)
Clinical training	No	172 (44%)
	Yes	216 (56%)
Medical problems	0-1	344 (89%)
	2-3	36 (9%)
	>3	8 (2%)
Rank	Enrolled nurse	0 (0%)
	Registered nurse	354 (91%)
	Advanced practice nurse	34 (9%)
	Nursing officer	0 (0%)
Years of experience in nursing	<2	98 (0%)
	2-5	74 (19%)
	>5	216 (56%)

### Survey Results and Implications

All of the correlation coefficients of the hypotheses were significant (*P* < .05), except for hypothesis 2 (*r* = .004, *P* < *.05*) (see Table 4). This implies that perceived ease of use of the Web 2.0 tools was not significant in predicting attitude toward their adoption. Therefore, hypothesis number H2 was rejected.

**Table 4:** Hypothesis testing results

Hypothesis	Content	Correlation Coefficient (Critical Value *r* = .08, *P* < .05)	Significance (Critical Value *t*_386_ = 1.65, *P* < .05)	Results
H1	Perceived usefulness of Web 2.0 tools is positively correlated with attitude toward its adoption.	.69	18.62	Accepted
H2	Perceived ease of use of Web 2.0 tools is positively correlated with attitude toward its adoption.	.004	Nil	Not Accepted
H3	Relative advantage of using Web 2.0 tools is positively correlated with attitude toward its adoption.	.64	16.53	Accepted
H4	Compatibility of using Web 2.0 tools is positively correlated with attitude toward its adoption.	.59	14.46	Accepted
H5	Peers’ attitude toward using Web 2.0 tools is positively correlated with subjective norm.	0.47	10.31	Accepted
H6	Senior management’s attitude toward using Web 2.0 tools is positively correlated with subjective norm.	0.24	4.835	Accepted
H7	Company’s attitude toward using Web 2.0 tools is positively correlated with subjective norm.	0.45	9.95	Accepted
H8	Resource facilitating conditions of Web 2.0 tools are positively correlated with perceived behavioral control.	.41	8.85	Accepted
H9	Technology facilitation conditions for using Web 2.0 tools are positively correlated with perceived behavioral control.	.69	18.78	Accepted
H10	Attitude toward Web 2.0 tools adoption is positively correlated with behavioral intention.	.72	20.20	Accepted
H11	Subjective norm concerning Web 2.0 tools adoption is positively correlated with behavioral intention.	.45	9.81	Accepted
H12	Perceived behavioral control to Web 2.0 tools adoption is positively correlated with behavioral intention.	.58	14.02	Accepted
H13	Behavioral intention toward Web 2.0 tools adoption is positively correlated with usage behavior.	0.60	14.77	Accepted

## Discussion

The first set of hypotheses showed that perceived usefulness (*r* = .69, *P* *<* *.05*), relative advantage (*r* = .64, *P* < .05), and compatibility (*r* = .59, *P* < .05) are positively correlated with attitude. The significance of the correlations between attitude and perceived usefulness (*t =* 18.62, *P* < .05), relative advantage (*t* = 16.53, *P <* .05), and compatibility (*t* = 14.46, *P* < .05) are high. This is because adopting Web 2.0 tools is not an objective decision but depends on how beneficial and useful [36,37] these tools will be to the nurses. In addition, the compatibility of Web 2.0 tools is also important to changing the actual behavior of nurses because of nurses’ concerns regarding whether the virtual environment of Web 2.0 tools can support knowledge sharing, learning, and social interaction in the traditional way. However, perceived ease of use is not a concern since most individuals have experience using Web 2.0 tools such as blogs and RSS or have used Internet technology in wired or wireless environments via personal desktops, notebooks, shopping kiosks, or mobiles.

The testing of the second set of hypotheses revealed that peer (*r* = .47, *P* <.05), senior management (*r* = .24*,* *P* < .05), and hospital influences (*r* = .45*, P* < .05) are positively correlated with subjective norm. The significance of the correlations between subjective norm and peer (*t* = 10.31, *P* < .05), senior management (*t* = 4.83, *P* < .05), and hospital (*t* = 9.95, *P* < .05) influences and are high. Peer and hospital influences are more significant than senior management influence. This can be explained by the fact that Web 2.0 is a virtual environment for the community, and a virtual community cannot be formed without peer participation. Thus, peer participation in activities over the Web 2.0 platform for knowledge sharing and social interaction significantly influences nurses’ decision to adopt it. On the other hand, since there may be some patient data privacy and confidentiality issues regarding the use of Web 2.0 tools for knowledge sharing [38], other issues important to nurses’ decisions are hospital policy, regulations, and guidance on the use of Web 2.0 tools. Most importantly, the hospital always plays a leadership role in promoting and supporting nurses’ adoption of new technology; thus, hospitals’ leadership and support in constructing a Web 2.0 environment for knowledge sharing, learning, social interaction, and the production of collective intelligence are important to their decision. Therefore, the hospital’s attitude is a major concern of nurses related to the adoption of Web 2.0 tools. In addition, senior management influence is also slightly relevant to nurses’ decisions because nurses require the support and encouragement of senior management to improve their nursing knowledge and learning. In summary, it can be concluded that peer participation and hospital support with policy and regulation on the use of Web 2.0 tools are the primary factors influencing their adoption by nurses and that senior management encouragement and support are secondary concerns.

The testing of the third set of hypotheses showed that the perceived behavioral control of human beings is positively correlated with resource (*r* = .41, *P* < .05) and technological conditions (*r* = .69, *P* < .05). The *t* value of the technology facilitating conditions (*t* = 18.78, *P* < .05) is higher than that of the resource facilitating conditions (*t* = 8.85, *P <* .05). This can be explained by the fact that nurses are mostly concerned about the availability of technology since Web 2.0 tools are new technology. Nurses are concerned about how and whether Web 2.0 functions can be accessed and used in Internet resources (eg, RSS feed reader) or their computing/mobile platform. This depends on the technology development of the Internet content or service providers or the technology infrastructure of the hospital environment [37]. By contrast, resource facilitating conditions such as money and time are less important to nurses when compared with technology facilitating conditions. 

Testing the last set of hypotheses showed that usage behavior (*r* = .60, *P* < .05) is positively correlated with behavioral intention. Behavioral intention is positively correlated with attitude (*r* = .72*,* *P* < .05), subjective norm (*r* = .45*,* *P* < .05), and perceived behavioral control (*r* = .58, *P* < .05). The significance of the correlation between usage behavior and behavioral intention (*t* = 14.77, *P* < .05) is high. The significance of the constructs between behavioral intention and attitude (*t* = 20.20, *P <* .05), perceived behavioral control (*t* = 14.02, *P* < .05), and subjective norm (*t* = 9.81, *P* < .05) are in descending order. The result of testing the hypothesis regarding usage behavior is similar to the finding of Ajzen [26] that the three constructs are also correlated with behavioral intention. Thus, it can be concluded that the significant priorities of nurses’ concerns regarding the adoption of Web 2.0 tools are attitude, perceived behavioral control, and subjective norm.

In conclusion, the primary concerns regarding the adoption of Web 2.0 tools are usefulness, advantages, compatibility, and technology availability and the secondary concerns are resource facilitating conditions and peer, hospital, and senior management attitude. The implication, then, is that health policy makers should make more effort to illustrate the usefulness, advantages, and compatibility of the application of Web 2.0 tools for knowledge sharing, learning, social interaction, and the production of collective intelligence and ensure that the technology is available to nurses. The other work for policy makers is to take a leadership role in promoting and supporting the adoption of Web 2.0 tools in the hospital environment and encouraging nurses to adopt Web 2.0 tools with their peers and senior management. Other resources such as money, time, and trainers can be subsidized or provided by the hospital authority.

Because physicians, nurses, and other health care professionals have close interaction, collaboration, and communication with each other on medical assessment, patient care and therapy, then knowledge sharing, learning, social interaction, and the production of collective intelligence are important for them to improve their skills and deliver a higher quality of medical service. Since Web 2.0 tools provide a platform to connect all these professionals together for knowledge sharing, learning, social interaction, and the production of collective intelligence, health policy makers can extend the use of Web 2.0 tools to these professionals. Therefore, using the DTPB can help them to identify their concerns regarding the adoption of Web 2.0 tools and to define strategies for promoting Web 2.0 in the whole hospital environment.

## Multimedia Appendix 1

Questionnaire for adoption of Web 2.0 tools for knowledge sharing, learning, social interaction, and collective intelligence.

## Abbreviations

DTPB: decomposed theory of planned behavior
IP: Internet protocol
IT: information technology
RSS: really simple syndication
TAM: technology acceptance model
TPB: theory of planned behavior
TRA: theory of reasoned action
UB: usage behavior
