# Supplementary material for: Hospital-Based Nurses’ Perceptions of the Adoption of Web 2.0 Tools for Knowledge Sharing, Learning, Social Interaction and the Production of Collective Intelligence
Source: J Med Internet Res. 2011 Nov 11;13(4):e92. doi: 10.2196/jmir.1398 (PMC3222197; doi:10.2196/jmir.1398)
Supplement: Supplementary file 1 [file jmir_v13i4e92_app1.pdf]

Web 2.0 tools provide a knowledge sharing, learning, social interaction and the production of collective intelligence platform. Blogs, wikis, tags, social bookmarks, really simple syndication (RSS), and social networking software are the tools on the Web 2.0 platform. Through the Web 2.0 platform, people can communicate and interact with their friends or peers, share and exchange their knowledge and experience, and share their happiness and unhappiness with their peers and friends over the web. In this study, you are required to answer a series of questions in order for us to determine your attitude and intentions to use the Web 2.0 platform for knowledge sharing, learning, social interaction, and the production of collective intelligence in your work environment or social community.

Part I Rank the importance of the factors

Please circle the rating. All measures have a 6-point scale.

1. I believe that Web 2.0 software would not be difficult to use.	Disagree	1 2 3 4 5 6	Agree
2. I believe that it would be easy to use Web 2.0 software to make friends or share/acquire knowledge.	Unlikely	1 2 3 4 5 6	Likely
3. I think learning to operate or master Web 2.0 software would present no difficulty to me.	Disagree	1 2 3 4 5 6	Agree
4. Using Web 2.0 software would improve the efficiency of my learning.	Unlikely	1 2 3 4 5 6	Likely
5. Using Web 2.0 software would enhance the effectiveness of my learning.	Disagree	1 2 3 4 5 6	Agree
6. I think using Web 2.0 software would be as easy as using other Internet applications.	Disagree	1 2 3 4 5 6	Agree
7. I believe my past knowledge in using the Internet would help me to use Web 2.0 software.	Impossible	1 2 3 4 5 6	Possible
8. I think communicating and knowledge sharing through Web 2.0 software would give me more advantages than using the face-to-face social interaction approach.	Impossible	1 2 3 4 5 6	Possible
9. The traditional method of social interaction or knowledge sharing would be replaced with such Web 2.0 software.	Undesirable	1 2 3 4 5 6	Desirable

10.	I think it would be _____ to use Web 2.0 software to communicate and share knowledge with other nurses rather than using the face-to-face approach.	Bad	1 2 3 4 5 6	Good
11.	Using Web 2.0 software would allow me to acquire nursing knowledge more quickly.	Disagree	1 2 3 4 5 6	Agree
12.	I think my future usage of Web 2.0 software is ____ if Web 2.0 software is developed in my working environment or social community.	Infrequent	1 2 3 4 5 6	Frequent
13.	If Web 2.0 software is ready to be used in my working environment or social community, the commitment to using Web 2.0 software is _________.	Weak	1 2 3 4 5 6	Strong
14.	If Web 2.0 software is ready to be used in your working environment or social community, how likely is it that you will use it?	Unlikely	1 2 3 4 5 6	Likely
15.	Your superior thinks Web 2.0 software will facilitate your work, knowledge, or social interaction, so you use it.	Disagree	1 2 3 4 5 6	Agree
16.	If most of your colleagues thought Web 2.0 software was useful for acquiring knowledge or social interaction, you would use it.	Disagree	1 2 3 4 5 6	Agree
17.	If the hospital expected everyone to use Web 2.0 software for social communication and knowledge sharing, you would do so.	Disagree	1 2 3 4 5 6	Agree
18.	I would spare time, money and effort to learn how to use Web 2.0 software.	Unlikely	1 2 3 4 5 6	Likely
19.	I think I would have enough knowledge to manage Web 2.0 software.	Disagree	1 2 3 4 5 6	Agree
20.	I believe that, with the existing technology, it is possible to implement and use this kind of Web 2.0 software.	Disagree	1 2 3 4 5 6	Agree
21.	I believe that using Web 2.0 software would be completely within my control.	Disagree	1 2 3 4 5 6	Agree

Part II Demographic Data

Fill in your demographic information.

1. Age: __21-30 __31-40 __41-50 __51-60 __>60
2. Gender: __Female __Male
3. Marital status: ___Single __Married
4. Educational level: __Sub-degree __ Bachelor’s __ Master’s
5. Continuous education: __No __Yes
6. Clinical training: __No __Yes
7. Medical problems: __0-1 __2-3 __>3
8. Rank: __Enrolled Nurse __Registered Nurse __Advanced Practice Nurse
   __Nurse Officer
9. Years of experience in nursing: __<2 __2-5 __>5

Abbreviations

DTPB: decomposed theory of planned behavior
IT: information technology
RDD:
RSS: Really Simple Syndication
TAM: technology acceptance model
TPB: theory of planned behavior
TRA: theory of reasoned action
